# Multiple conformational states in retrospective virtual screening – homology models vs. crystal structures: beta-2 adrenergic receptor case study

**DOI:** 10.1186/s13321-015-0062-x

**Published:** 2015-04-09

**Authors:** Stefan Mordalski, Jagna Witek, Sabina Smusz, Krzysztof Rataj, Andrzej J Bojarski

**Affiliations:** Department of Medicinal Chemistry, Institute of Pharmacology, Polish Academy of Sciences, 12 Smętna Street, Kraków, 31-343 Poland; Faculty of Chemistry, Jagiellonian University, 3 R. Ingardena Street, Kraków, 30-060 Poland

**Keywords:** Crystal structures, Homology models, Virtual screening, Structural Interaction Fingerprints, Ensemble of receptors

## Abstract

**Background:**

Distinguishing active from inactive compounds is one of the crucial problems of molecular docking, especially in the context of virtual screening experiments. The randomization of poses and the natural flexibility of the protein make this discrimination even harder. Some of the recent approaches to post-docking analysis use an ensemble of receptor models to mimic this naturally occurring conformational diversity. However, the optimal number of receptor conformations is yet to be determined.

In this study, we compare the results of a retrospective screening of beta-2 adrenergic receptor ligands performed on both the ensemble of receptor conformations extracted from ten available crystal structures and an equal number of homology models. Additional analysis was also performed for homology models with up to 20 receptor conformations considered.

**Results:**

The docking results were encoded into the Structural Interaction Fingerprints and were automatically analyzed by support vector machine. The use of homology models in such virtual screening application was proved to be superior in comparison to crystal structures. Additionally, increasing the number of receptor conformational states led to enhanced effectiveness of active vs. inactive compounds discrimination.

**Conclusions:**

For virtual screening purposes, the use of homology models was found to be most beneficial, even in the presence of crystallographic data regarding the conformational space of the receptor. The results also showed that increasing the number of receptors considered improves the effectiveness of identifying active compounds by machine learning methods.

**Graphical abstract:**
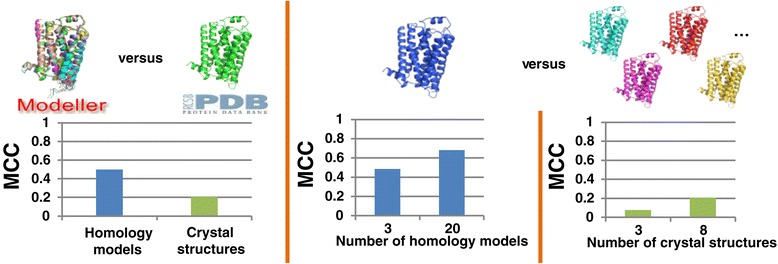
Comparison of machine learning results obtained for various number of beta-2 AR homology models and crystal structures.

**Electronic supplementary material:**

The online version of this article (doi:10.1186/s13321-015-0062-x) contains supplementary material, which is available to authorized users.

## Background

G protein-coupled receptors (GPCRs) constitute a large superfamily of signaling proteins that share a common topology of 7 transmembrane (7TM) helices and transduce signals across the cell membrane. Because GPCRs are responsible for most of a cell’s communication with its environment, their malfunctions are associated with various disease states, mainly those related to the central nervous system (CNS). For this reason, GPCRs are a very important target base for drugs [1-3].

The beta-2 adrenergic (B2AR) receptor, the subject of this case study, is representative of the class A GPCRs and is involved in mediating the relaxation of smooth muscle, glycogenolysis and glucogenesis in the liver and regulation of the metabolism of cells in skeletal muscle. Β2AR is also responsible for increased cardiac output, facilitation of the release of neurotransmitters, and regulation of various other physiological processes [4-7]. B2AR is also one of the most studied 7TM structures; it was first crystalized in 2007 [8], and as of November 2014, 16 crystals with a variety of structurally and functionally unique ligands are available via the Protein Data Bank (PDB), making this receptor a strong base for *in silico* structural studies.

Our previous study applying Machine Learning (ML) to post-docking analysis used Structural Interaction Fingerprint (SIFt) profiles created upon three different crystalline conformations of receptors [9,10]. It showed the applicability of this approach to ligand-protein complexes evaluation for Virtual Screening (VS). In addition to the issue of the applicability of crystal structures in VS, this study also investigates the influence of the number of conformations used in per-ligand interaction profiles, for both crystal structures and homology models, on retrospective screening performance. The VS setup consisted of four groups of compounds: active, inactive, DUD (Directory of Useful Decoys) decoys, and random ZINC subsets; three sets of experiments were prepared to discriminate between active and inactive compounds from each of the decoy collections.

The support vector machine (SVM) was the classification algorithm chosen and the VS performance was measured with the Matthews Correlation Coefficient (MCC).

## Results and discussion

### Crystal structures vs. homology models

**Figure 1:**
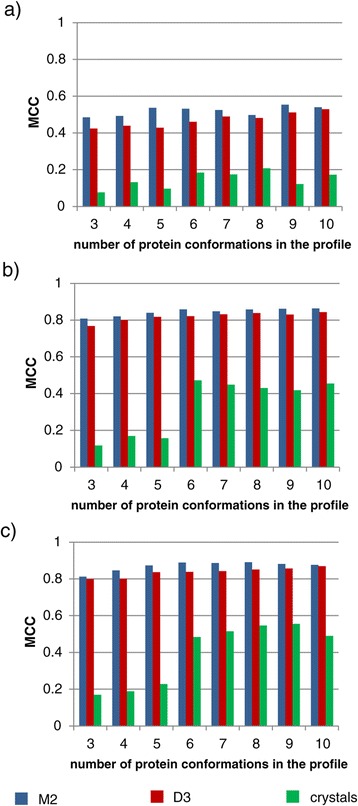
**Comparison of MCC values obtained in the ML-based experiments of docking results to homology models built on M2R and D3R template and crystal structures for discrimination between a) actives/true inactives, b) actives/DUDs, and c) actives/ZINC.** The figure presents the MCC values obtained for homology models of beta-2 adrenergic receptor (the best and the worst template) and for crystal structures of this receptor in experiments distinguishing the following class of compounds: **(a)** actives/true inactives, **(b)** actives/DUDs and **(c)** actives/ZINC.

Because the number of crystal templates used for homology models construction would affect the clarity of the presented results, the comparison of homology models and crystal structures is shown for the templates providing the best (M2R) and the worst (D3R) results (in terms of the discrimination between actives and true inactives) – Figure 1; the outcomes for the remaining templates are available in the Additional files section (Additional file 1: Figure S1). The use of vast numbers of templates for homology modeling follows the protocols used in previously published data and ensures maximum VS performance [11]. Due to a limited number of available crystal structures, the maximum number of receptor conformations in this comparison is restricted to 10 (starting from 3).

The results of retrospective VS (Figure 1) show that homology model-based screening significantly outperforms experiments conducted for the collection of crystal structures, with MCC improvement of 0.4 for the best set of conformations. In addition, all types of classification experiments (actives/true inactives, actives/DUDs, and actives/ZINC cmds) confirm this dependency. The MCC spreads for different templates were of little significance: variation between the best and the worst performing template ranged from 0.1 for actives/true inactives experiments to less than 0.05 for the other two VS scenarios.

For homology models, MCC values obtained for actives/true inactives discrimination were the lowest (~0.5 – 0.55). However, for actives/DUDs and actives/ZINC cmds classifications, MCC values exceeded 0.8, with a slight preference towards actives/ZINC experiments.

On the other hand, studies performed for crystal structures resulted in MCC of 0.2 for actives/true inactives (this best result was obtained for the SIFt profile composed of 8 receptor conformations), 0.47 for actives/DUDs (6 conformations) and 0.55 for actives/ZINC (9 conformations).

The obtained results show that the conformational flexibility provided by homology models allows for better accommodation of diverse ligands and therefore better screening performance in this interaction-centric type of experiments. Because crystal structures are limited in terms of chemical space of co-crystalized ligands, they are not yet able to provide a sufficient conformational landscape for efficient identification of active compounds.

### Influence of the number of considered conformations on screening performance for homology models

**Figure 2:**
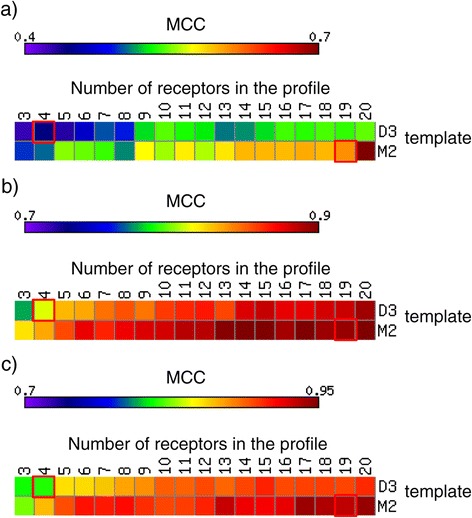
**MCC values obtained for various numbers of models included in the SIFt profile for models built on M2R and D3R templates for a) actives/true inactives, b) actives/DUDs, and c) actives/ZINC discrimination.** The figure presents the MCC values obtained for various numbers of models included in the SIFt profile for homology models constructed on M2R and D3R templates in the form of the heat map for **a)** actives/true inactives, **b)** actives/DUDs, **c)** actives/ZINC cmds discrimination. Red frames indicate the step when the best model in terms of the AUROC value was added to the profile.

Due to the substantial amount of data, a detailed analysis was conducted only for the best performing set of SVM parameters, in terms of MCC value, and also for the best and the worst template only (M2R and D3R_,_ respectively); full datasets are included within the Additional files (Additional file 2: Figure S2). The MCC values related to different numbers of considered conformations are presented in Figure 2.

**Figure 3:**
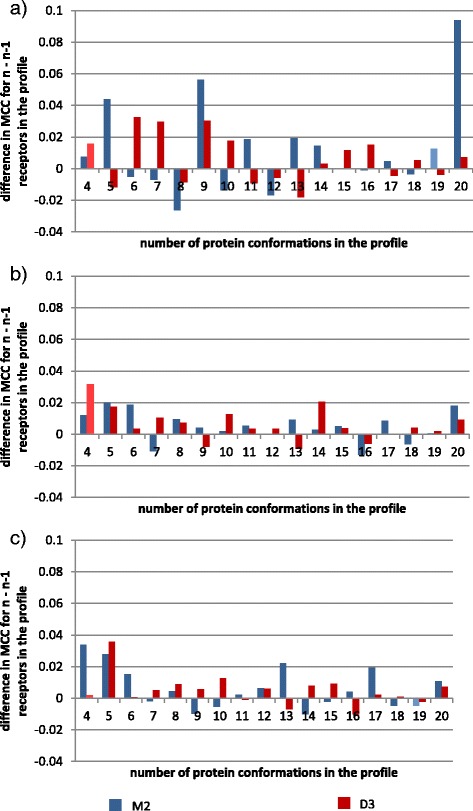
**Difference in MCC caused by the inclusion of additional receptors in the profile for a) actives/true inactives, b) actives/DUDs, c) actives/ZINC cmds discrimination.** The figure presents the changes in MCC obtained after the inclusion of additional receptors in the SIFt profile for homology models constructed on M2R and D3R templates. The introduction of the best model in terms of the AUROC values is indicated by lighter colour.

In addition, differential graphs illustrating MCC change after including subsequent models (adding one-by-one- from 3 to 20 forming at the end 20-models-based profile) were prepared (Figure 3, Additional file 3: Figure S3). In each case, the addition of the model that was characterized by the highest area under the ROC curve (AUROC) at the stage of models evaluation was highlighted – in Figure 2 by red frame, in Figure 3 by the application of brighter colour.

The general outcome emerging from the results (Figure 2; Figure 3) aligns with the results obtained for the comparison of crystal structures with homology models. An increased number of conformations included in a SIFt profile leads to an improvement of VS performance, however, for some isolated cases, the contribution of subsequent models may be negative. The number of models providing the highest MCC was 20 in the majority of cases (as shown in Table 1), and the worst performing set of conformations was 3 for all but two sets of models. The improvement of MCC was not linear; however, its values were noticeably lower for a low number of conformations considered.

**Table 1:** **The optimal and the worst number of models included in the SIFt profiles in terms of classification effectiveness**

**Template**	**Actives vs. true inactives**		**Actives vs. DUD**		**Actives vs. ZINC**	
	**Optimal number of receptors**	**The number of receptors with min MCC**	**Optimal number of receptors**	**The number of receptors with min MCC**	**Optimal number of receptors**	**The number of receptors with min MCC**
5-HT1BR	20	4	18	4	8	3
5-HT2BR	18	3	18	3	19	3
A2AR	19	3	20	3	20	3
Beta1R	17	10	20	6	20	3
CXCR4R	10	4	15	3	19	6
D3R	20	3	20	3	15	3
H1R	20	5	20	3	20	4
M2R	20	3	20	3	20	3
M3R	20	3	20	3	18	3
Crystal structures	8	3	6	3	9	3

MCC fluctuations occurring in the actives/true inactives classification stage of the experiment were the highest out of all three scenarios. This scenario also had several situations where additional considered conformations lowered the screening performance. On the other hand, filtering actives against DUD and random ZINC decoys led to a clear dependency between MCC and the number of models: the higher the number of models included in the profile, the higher the MCC values.

The impact of receptors bearing the highest AUROC values during the model selection step (conformation 4 and 19 for D3R and M2R templates, respectively) proves that the performance of individual homology models has little influence on the obtained results and, in some cases (conformation 19 based on M2R in screening against ZINC subset – Figure 2c), can even lower VS performance.

Although the MCC changes induced by including new conformations to the ligand profiles seem to be negligible, the cumulative effect for VS experiments leads to a significant improvement of screening performance by up to 20% (Additional file 4: Figure S4). Interestingly, the absolute values of MCC difference oscillated at approximately 0.1, regardless of the scheme of the experiment.

### Influence of the number of considered conformations on screening performance for crystal structures

**Figure 4:**
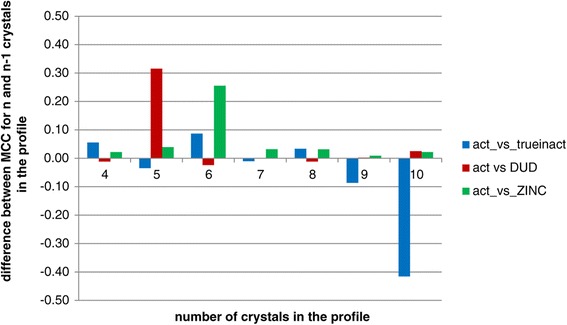
**Changes in MCC after the inclusion of additional crystals in the profile.** The figure presents the changes in MCC values caused by the addition of subsequent crystals to the profile (adding one-by-one- from 3 to 10 forming at the end 10-crystals-based profile) in experiments where docking was performed to crystal structures of beta-2 adrenergic receptor.

The results show that the experiments using multiple crystalline conformations are significantly more prone to screening performance fluctuations (Figure 4). The amplitude of these fluctuations ranges from a 0.3 improvement to a 0.4 decrease in terms of MCC value. This variation of the results obtained for crystal structures is connected with the specificity of the individual crystallized with the proteins. The drop in MCC, observed after adding the last two conformations (3P0G [12] and 3PDS [13]), is a consequence of the crystals being agonist bound. The conformation of an activated GPCR results in limited ligand accessible volume, significantly reducing the quality of docking results. Following this lead, the influence of activation state of the crystal template on classification efficiency was also examined for beta-2 homology models constructed on activated and deactivated M2R template. The direction of changes when adding subsequent SIFts to the profile was preserved but the results were slightly (~2-3%) better for models constructed on deactivated template (Additional file 5: Figure S5).

## Conclusions

In this study we compared the performance of the collection of crystal structures with corresponding sets of homology models in retrospective VS experiments designed to consider multiple conformations of a target receptor. The results demonstrated that the bundle of homology models significantly outperformed the crystalline-based approach in terms of MCC, which agrees with results from previous reports [14]. The main reason behind this difference in screening effectiveness is the limited conformational space of the crystal structures, which is a consequence of adaptation to the co-crystalized ligands, thus biasing the conformation of the complex. Shallow conformational landscapes of the crystal structures of the receptors are also caused by low structural diversity of the crystalline ligands, limiting the possible spatial orientations of residues.

The second component of this research investigated the effect of increasing the number of considered conformations. The conclusion emerging from all schemes of experiments (screening active compounds against truly inactive, DUD, and ZINC decoys) is that high coverage of the conformational space of the receptor models leads to more effective screening. A probable reason behind this observation is that the inclusion of more conformations into a docking protocol neglects the fluctuations of docking poses and provides a more coherent binding mode for a given ligand, therefore enabling a clearer discrimination between active and inactive compounds. Extending the population of conformations would most likely increase the MCC up to the limit defined by the number of compounds that were not docked into any receptor model, yet the increasing computational cost of such tests may render the results not worth the effort. Although there is no actual boundary for the number of conformations to include, the results shown here prove that three models/crystals leave sufficient space to improve VS performance.

## Methods

To maintain the coherence of the ligand data, the compounds of known activity (divided into sets ‘actives’ and ‘true inactives’) were extracted using a strict protocol. All structures with verified activity towards the B2AR were selected from the ChEMBL database [15]. Only those compounds whose activity was quantified in K_i_ or IC_50_ (with the assumption that K_i_ = IC_50_/2) and that were tested on human cloned, rat cloned, or native receptors were taken into account. A compound was considered active when the K_i_ value assigned to it was lower than 100 nM, and the compound was considered inactive when this activity parameter was higher than 1000 nM. The compounds were clustered with Canvas [16], and the number of clusters was set to approximately 30% of the total number of compounds from a particular group. Cluster centroids were used for the primary evaluation of homology models. In addition, two sets of decoy compounds were generated, one following the DUD methodology [17] and one random subset of the ZINC database [18]. Both of the decoy collections contained 2000 compounds; DUDs were randomly picked to narrow the count of the set.

The homology models of the B2AR were constructed. Nine crystal templates were used for this purpose: serotonin receptors 5-HT1B and 5-HT2B, adenosine receptor A2A, adrenergic receptor beta-1, chemokine receptor CXCR4, dopamine receptor D3, histamine receptor H1, and muscarinic receptors M2 and M3 (Table 2). The sequence alignment was performed manually and only for the transmembrane helices. Loops were not modelled. For each template, 20 models were generated with Modeller 9v13 software [19] and were evaluated by AUROC in the docking of cluster centroids from actives and true inactives sets (Table 3). Additional homology models were also prepared in the same manner for deactivated M2R structure (4MQS).

**Table 2:** **Crystal structures used as templates for homology modeling of beta-2 adrenergic receptor**

**Template**	**PDB ID**	**Resolution [Å]**
5-HT1BR	4IAR [23]	2.70
5-HT2BR	4IB4 [24]	2.70
A2AR	3QAK [25]	2.71
Beta1R	2Y00 [26]	2.50
CXCR4R	3OE0 [27]	2.90
D3R	3PBL [28]	2.89
H1R	3RZE [29]	3.10
M2R	3UON [30]	3.00
M3R	4DAJ [31]	3.40

**Table 3:** **Number of compounds used for the preevaluation of homology models**

**Group of compounds**	**All**	**Number of clusters**	**Number of centroids after Ligprep**
Actives	271	81	103
Inactives	324	97	173

The three dimensional structures of the compounds, along with protonation states and atom types, were assigned with LigPrep software [20]. For some compounds, several protonation states were generated what increased the initial number of instances. The docking was performed with GLIDE 5.0, with the number of output poses limited to one for both homology models (Table 4), and a collection of B2AR crystal structures (Table 5).

**Table 4:** **Compound counts for retrospective screening scenarios**

**Group of compounds**	**Total number of compounds**	**Number of compounds after Ligprep**
actives	271	550
Inactives	324	601
DUDs	2000	2526
ZINC	2000	2557

**Table 5:** **Crystal structures of beta-2 adrenergic receptor used in the study**

**PDB ID**	**Resolution [Å]**
2RH1	2.40
3D4S	2.80
3NY8	2.84
3NY9	2.84
3NYA	3.16
3KJ6	3.40
2R4R	3.40
2R4S	3.40
3P0G	3.50
3PDS	3.50

After this initial models evaluation, all compounds from a particular group of molecules (actives, true inactives, DUDs, and ZINC) were docked into the constructed homology models and crystal structures. Ligand-receptor complexes received from the docking procedure were represented by the Structural Interaction Fingerprint [9] which have a type of a binary string that describes the interaction of a ligand with each of the amino acids of the protein; the string is divided into nine-bit chunks that refer to particular amino acid residues. The type of interactions that are taken into account include the presence of any interaction, an interaction with the main chain, an interaction with a side chain, a polar interaction, a hydrophobic interaction, a hydrogen bond acceptor, a hydrogen bond donor, an aromatic interaction, and a charged bond.

For each compound that had at least one pose in a population of receptor conformations, the SIFt profile was calculated. On each position in the string, the values were averaged over all models/crystal structures for the given conformational landscape considered (per ligand SIFt profile). The number of receptor conformations used in the experiments ranged from 3 to 20 (10 for the crystal structures).

The per ligand SIFt profiles were input for machine learning experiments conducted with the use of the WEKA package [21]. The task of the ML algorithm was to distinguish active from inactive or decoy compounds. Support vector machines algorithm [22] was used as a classification method with linear function as a kernel. This model was developed by Vapnik [22] with a core concept of seeking the hyperplane separating the binary-labeled data with the maximum possible margin. This can be written as the following optimization problem:

$$ \underset{w,\kern0.1em b}{ \min \mathrm{imize}}\kern0.1em \frac{1}{2}\parallel w{\parallel}^2+C\kern0.1em \sum_i^N\kern0.1em {\xi}_i $$

$$ \mathrm{subject}\kern0.5em \mathrm{t}\mathrm{o}\kern0.5em {y}_i\left(\left\langle w,\kern0.1em {x}_i\right\rangle -b\right)\kern0.5em \ge \kern0.5em 1\kern0.5em -\kern0.5em {\xi}_i\kern0.5em -\kern0.5em \mathbf{v}\mathbf{a}\mathbf{r}\left({\boldsymbol{\alpha}}_{\boldsymbol{i}}\right){\boldsymbol{\xi}}_{\boldsymbol{i},}i\kern0.5em =\kern0.5em 1,\kern0.1em \dots \kern0.1em ,\kern0.1em N $$

with *w* being the normal vector to the hyperplane and *y*_i_ being the class to which the particular example is assigned (in case of binary labeled data, *y*_*i*_ ∈ {− 1, + 1}). *C* is the parameter that controls the tradeoff between the correct classification and large margin.

However, in real applications, the data are not usually linearly separable, and the application of the kernel trick is required. In our paper, the linear kernel ($K\left({x}_i,\kern0.1em {x}_j\right)\kern0.5em =\kern0.5em \left\langle {x}_i,{x}_j\right\rangle \kern0.5em =\kern0.5em {\displaystyle \sum_{l=1}^d}\kern0.5em {x_i}_l{x}_{jl}$ for d-dimensional feature space) was applied.

The original optimization problem is transformed to the dual form with the use of Lagrange’s multipliers *α*_*i*_:

$$ \underset{\alpha }{ \max \mathrm{imize}}\kern0.5em {\displaystyle \sum_{i=1}^N{\alpha}_i}\kern0.5em -\kern0.5em \frac{1}{2}\kern0.5em {\displaystyle \sum_{i,\kern0.1em j}^N{a}_i{a}_j{y}_i{y}_jK\left({x}_i,{x}_j\right)} $$

$$ \mathrm{subject}\kern0.5em \mathrm{t}\mathrm{o}\kern0.5em 0\kern0.5em \le \kern0.5em {\alpha}_i\kern0.5em \le \kern0.5em C,\kern0.5em i= 1,\dots, N $$

$$ \sum_{i=1}^N\kern0.5em {\alpha}_i{y}_i\kern0.5em =\kern0.5em 0 $$

*α*_*i*_ represents weights that are assigned to particular example *x*_*i*_ from the training data, and in the dual form, *C* constitutes the upper bound of *α*_*i*_ values.

The optimization of *C* values was performed (the following *C* values were checked: 0.01; 0.1; 1; 10; 100; 1 000; 10 000). The experiments were carried out in a 10-fold cross-validation mode.

The effectiveness of machine learning methods was measured with MCC being a balanced measure for such kind of experiments and expressed by the following formula:

$$ MCC=\frac{TP\cdot TN-FP\cdot FN}{\sqrt{\left(TP+FP\right)\cdot \left(TP+FN\right)\cdot \left(TN+FP\right)\cdot \left(TN+FN\right)}} $$

where:

- *TP* – number of true positives,
- *TN* – number of true negatives,
- *FP* – number of false positives
- *FN* – number of false negatives

## Additional files

Additional file 1: Figure S1. Comparison of MCC values obtained in the ML-based experiments for homology models and crystal structures for discrimination between a) actives/true inactives, b) actives/DUDs, and c) actives/ZINC. The figure presents the MCC values obtained for homology models of beta-2 adrenergic receptor (constructed on various templates) and for crystal structures of this receptor in experiments distinguishing the following class of compounds: (a) actives/true inactives, (b) actives/DUDs and (c) actives/ZINC. Additional file 2: Figure S2. MCC values obtained for various numbers of models included in the SIFt profile for models built on various templates for a) actives/true inactives, b) actives/DUDs, and c) actives/ZINC discrimination. The figure presents the MCC values obtained for various numbers of models included in the SIFt profile for homology models constructed on various templates in the form of the heat map for a) actives/true inactives, b) actives/DUDs, c) actives/ZINC cmds discrimination. Additional file 3: Figure S3. Difference in MCC caused by the inclusion of additional receptors in the profile for a) actives/true inactives, b) actives/DUDs, and c) actives/ZINC cmds discrimination. The figure presents the changes in MCC obtained after the inclusion of additional receptors in the SIFt profile for homology models. Additional file 4: Figure S4. Difference between the highest and the lowest MCC obtained for SIFt profiles construction for various numbers of conformations. The figure presents the scale of MCC changes associated with varying number of model conformations in the form of differences between the highest and the lowest. MCC values obtained for a given template/crystal structure. Additional file 5: Figure S5. Comparison of the actives/true inactives classification efficiency for beta-2 homology models constructed on activated and deactivated M2R template. The figure presents the differences between the results for beta-2 homology models that were constructed on crystal structure of the receptor with agonist (activated template) and on crystal structure in which the receptor was bound to antagonist (deactivated structure).

## Abbreviations

GPCRs: G protein-coupled receptors
7TM: 7 transmembrane
CNS: Central nervous system
B2AR: Beta-2 adrenergic
PDB: Protein data bank
SIFt: Structural interaction fingerprint
ML: Machine learning
VS: virtual screening
DUD: Directory of useful decoys
SVM: Support vector machine
MCC: Matthews correlation coefficient

## References

[1] Klabunde T, Hessler G (2002). Drug design strategies for targeting G-protein-coupled receptors. ChemBioChem.

[2] Lagerström MC, Schiöth HB (2008). Structural diversity of G protein-coupled receptors and significance for drug discovery. Nat Rev Drug Discov.

[3] Lundstrom K (2009). An overview on GPCRs and drug discovery: structure-based drug design and structural biology on GPCRs. Methods Mol Biol.

[4] Liggett SB (1999). Molecular and genetic basis of beta2-adrenergic receptor function. J Allergy Clin Immunol.

[5] McGraw DW, Liggett SB (2005). Molecular mechanisms of beta2-adrenergic receptor function and regulation. Proc Am Thorac Soc.

[6] Kolb P, Rosenbaum DM, Irwin JJ, Fung JJ, Kobilka BK, Shoichet BK (2009). Structure-based discovery of beta2-adrenergic receptor ligands. Proc Natl Acad Sci USA.

[7] Strosberg AD (1993). Structure, function, and regulation of adrenergic receptors. Protein Sci.

[8] Cherezov V, Rosenbaum DM, Hanson MA, Rasmussen SGF, Thian FS, Kobilka TS (2007). High-resolution crystal structure of an engineered human beta2-adrenergic G protein-coupled receptor. Science.

[9] Deng Z, Chuaqui C, Singh J (2004). Structural interaction fingerprint (SIFt): a novel method for analyzing three-dimensional protein-ligand binding interactions. J Med Chem.

[10] Witek J, Smusz S, Rataj K, Mordalski S, Bojarski AJ (2014). An application of machine learning methods to structural interaction fingerprints - a case study of kinase inhibitors. Bioorg Med Chem Lett.

[11] Rataj K, Witek J, Mordalski S, Kosciolek T, Bojarski AJ (2014). Impact of template choice on homology model efficiency in virtual screening. J Chem Inf Model.

[12] Rasmussen SGF, Choi H-J, Fung JJ, Pardon E, Casarosa P, Chae PS (2011). Structure of a nanobody-stabilized active state of the β(2) adrenoceptor. Nature.

[13] Rosenbaum DM, Zhang C, Lyons JA, Holl R, Aragao D, Arlow DH (2011). Structure and function of an irreversible agonist-β(2) adrenoceptor complex. Nature.

[14] Tang H, Wang XS, Hsieh JH, Tropsha A (2012). Do crystal structures obviate the need for theoretical models of GPCRs for structure-based virtual screening?. Proteins.

[15] Gaulton A, Bellis LJ, Bento AP, Chambers J, Davies M, Hersey A (2011). ChEMBL: a large-scale bioactivity database for drug discovery. Nucl Acids Res.

[16] Canvas, version 1.3, Schrödinger, LLC, New York, NY, 2010.

[17] Huang N, Shoichet BK, Irwin JJ (2006). Benchmarking sets for molecular docking. J Med Chem.

[18] Irwin JJ, Shoichet BK (2005). ZINC - a free database of commercially available compounds for virtual screening. J Chem Inf Model.

[19] Sali A, Blundell TL (1993). Comparative protein modelling by satisfaction of spatial restraints. J Mol Biol.

[20] LigPrep, version 2.5, Schrödinger, LLC, New York, NY, 2011.

[21] Hall M, Frank E, Holmes G, Pfahringer B, Reutemann P, Witten IH (2009). The WEKA data mining software: an update. SIGKDD Explorations.

[22] Cortes C, Vapnik V (1995). Support-vector networks. Mach Learn.

[23] Wang C, Jiang Y, Ma J, Wu H, Wacker D, Katritch V (2013). Structural basis for molecular recognition at serotonin receptors. Science.

[24] Wacker D, Wang C, Katritch V, Han GW, Huang X-P, Vardy E (2013). Structural features for functional selectivity at serotonin receptors. Science.

[25] Xu F, Wu H, Katritch V, Han GW, Jacobson KA, Gao Z-G (2011). Structure of an agonist-bound human A2A adenosine receptor. Science.

[26] Warne T, Moukhametzianov R, Baker JG, Nehmé R, Edwards PC, Leslie AGW (2011). The structural basis for agonist and partial agonist action on a β(1)-adrenergic receptor. Nature.

[27] Wu B, Chien EYT, Mol CD, Fenalti G, Liu W, Katritch V (2010). Structures of the CXCR4 chemokine GPCR with small-molecule and cyclic peptide antagonists. Science.

[28] Chien EYT, Liu W, Zhao Q, Katritch V, Han GW, Hanson MA (2010). Structure of the human dopamine D3 receptor in complex with a D2/D3 selective antagonist. Science.

[29] Shimamura T, Shiroishi M, Weyand S, Tsujimoto H, Winter G, Katritch V (2011). Structure of the human histamine H1 receptor complex with doxepin. Nature.

[30] Haga K, Kruse AC, Asada H, Yurugi-Kobayashi T, Shiroishi M, Zhang C (2012). Structure of the human M2 muscarinic acetylcholine receptor bound to an antagonist. Nature.

[31] Kruse AC, Hu J, Pan AC, Arlow DH, Rosenbaum DM, Rosemond E (2012). Structure and dynamics of the M3 muscarinic acetylcholine receptor. Nature.

