# Supplementary material for: Multiple conformational states in retrospective virtual screening – homology models vs. crystal structures: beta-2 adrenergic receptor case study
Source: J Cheminform. 2015 Apr 9;7:13. doi: 10.1186/s13321-015-0062-x (PMC4420846; doi:10.1186/s13321-015-0062-x)
Supplement: Additional file 1: Figure S1. — Comparison of MCC values obtained in the ML-based experiments for homology models and crystal structures for discrimination between a) actives/true inactives, b) actives/DUDs, and c) actives/ZINC. The figure presents the MCC values obtained for homology models of beta-2 adrenergic receptor (constructed on various templates) and for crystal structures of this receptor in experiments distinguishing the following class of compounds: (a) actives/true inactives, (b) actives/DUDs and (c) actives/ZINC. [file 13321_2015_62_MOESM1_ESM.pdf]

Figure S1. Comparison of MCC values obtained in the ML-based experiments for homology models and crystal structures for discrimination between a) actives/true inactives, b) actives/DUDs, and c) actives/ZINC.

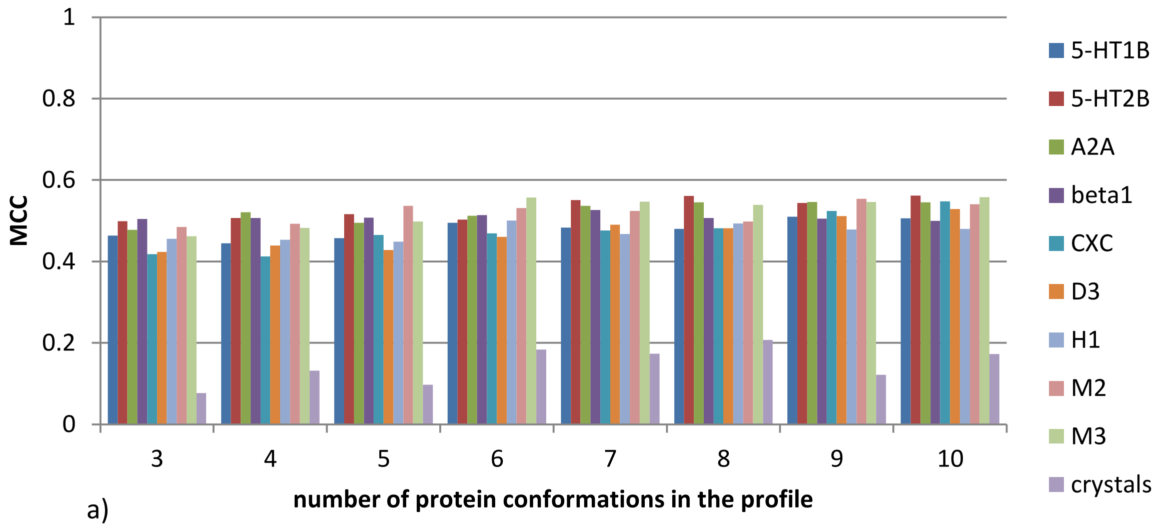

a)

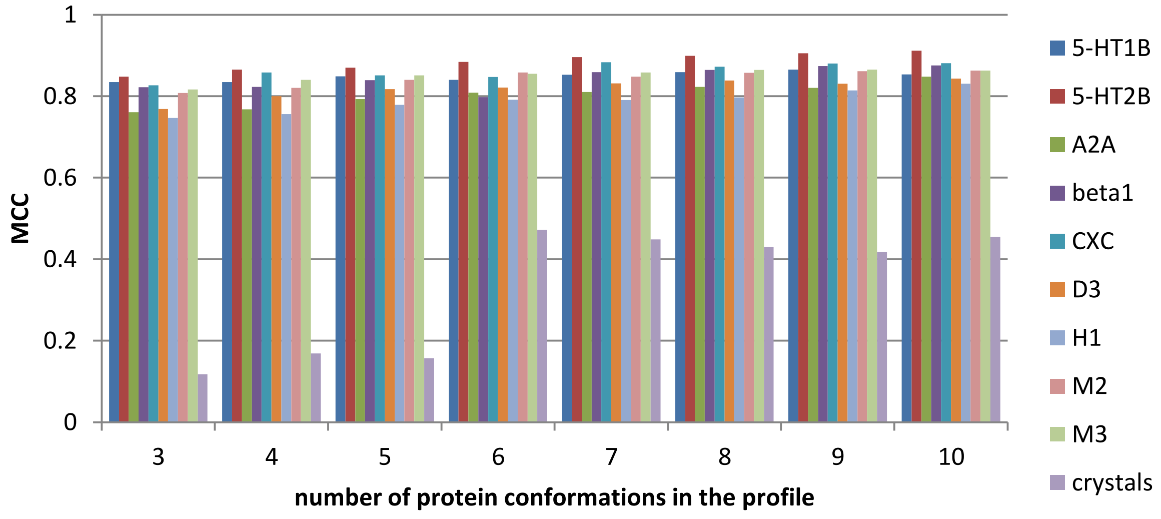

b)

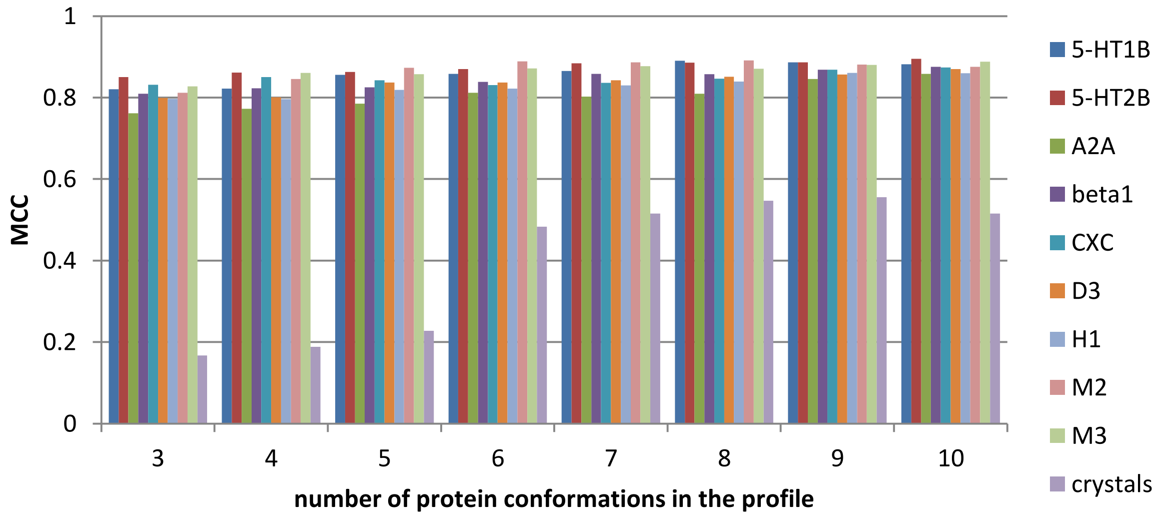

c)
