# Supplementary material for: Multiple conformational states in retrospective virtual screening – homology models vs. crystal structures: beta-2 adrenergic receptor case study
Source: J Cheminform. 2015 Apr 9;7:13. doi: 10.1186/s13321-015-0062-x (PMC4420846; doi:10.1186/s13321-015-0062-x)
Supplement: Additional file 2: Figure S2. — MCC values obtained for various numbers of models included in the SIFt profile for models built on various templates for a) actives/true inactives, b) actives/DUDs, and c) actives/ZINC discrimination. The figure presents the MCC values obtained for various numbers of models included in the SIFt profile for homology models constructed on various templates in the form of the heat map for a) actives/true inactives, b) actives/DUDs, c) actives/ZINC cmds discrimination. [file 13321_2015_62_MOESM2_ESM.pdf]

Figure S2. MCC values obtained for various numbers of models included in the SIFt profile for models built on various templates for a) actives/true inactives, b) actives/DUDs, and c) actives/ZINC discrimination

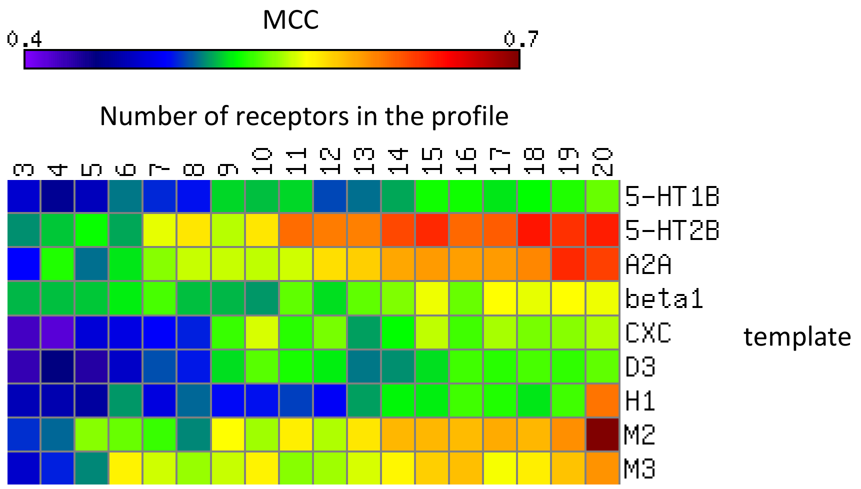

a)

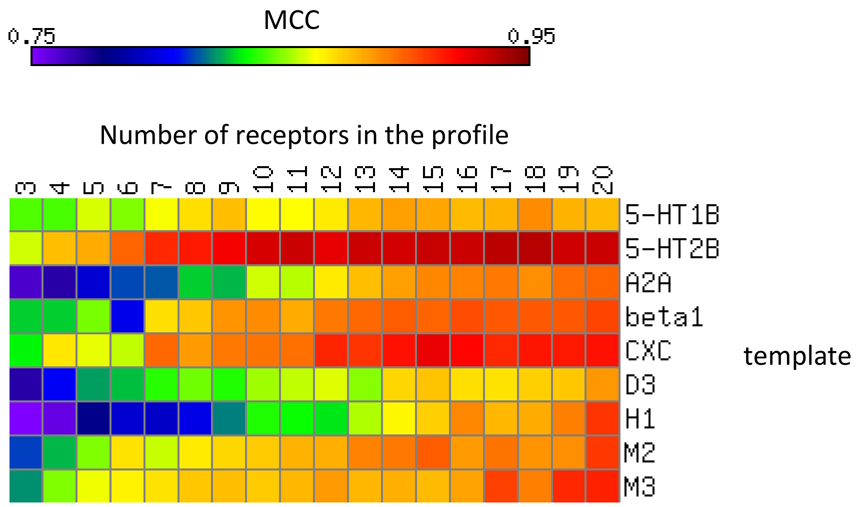

b)

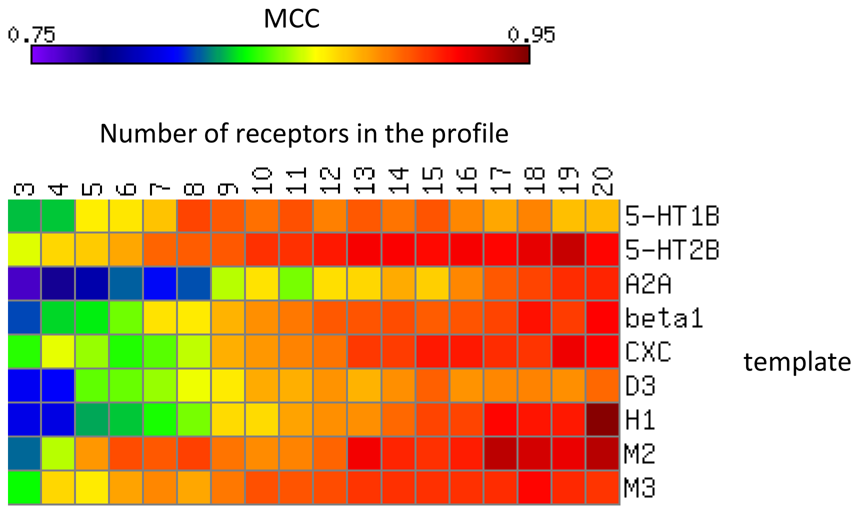

c)
