# Supplementary material for: Multiple conformational states in retrospective virtual screening – homology models vs. crystal structures: beta-2 adrenergic receptor case study
Source: J Cheminform. 2015 Apr 9;7:13. doi: 10.1186/s13321-015-0062-x (PMC4420846; doi:10.1186/s13321-015-0062-x)

Figure S3. Difference in MCC caused by the inclusion of additional receptors in the profile for a) actives/true inactives, b) actives/DUDs, c)actives/ZINC cmds discrimination.

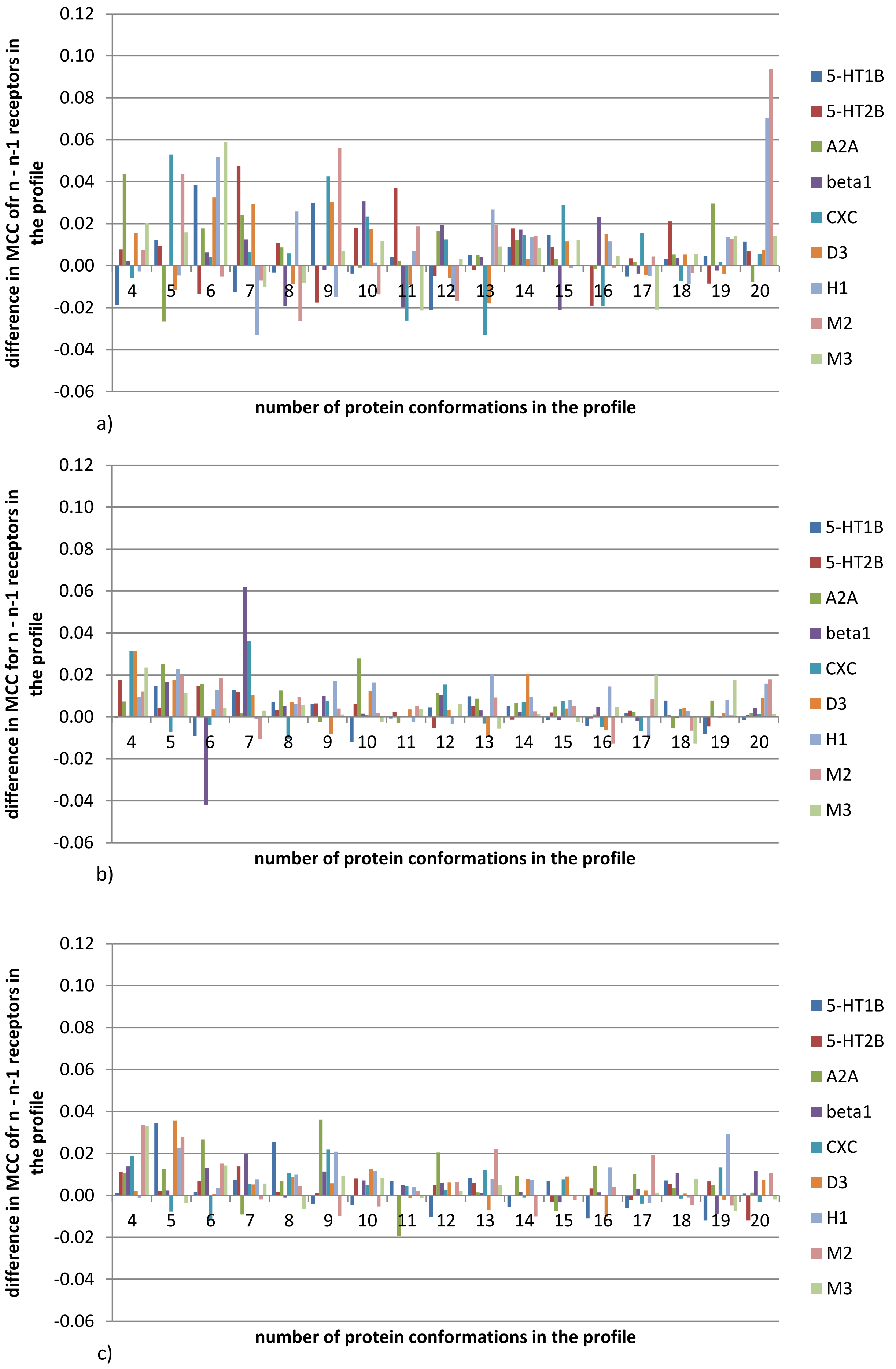

Supplement: Additional file 3: Figure S3. — Difference in MCC caused by the inclusion of additional receptors in the profile for a) actives/true inactives, b) actives/DUDs, and c) actives/ZINC cmds discrimination. The figure presents the changes in MCC obtained after the inclusion of additional receptors in the SIFt profile for homology models. [file 13321_2015_62_MOESM3_ESM.pdf]
