# Supplementary figures and images for: Multiple conformational states in retrospective virtual screening – homology models vs. crystal structures: beta-2 adrenergic receptor case study
Source: J Cheminform. 2015 Apr 9;7:13. doi: 10.1186/s13321-015-0062-x (PMC4420846; doi:10.1186/s13321-015-0062-x)

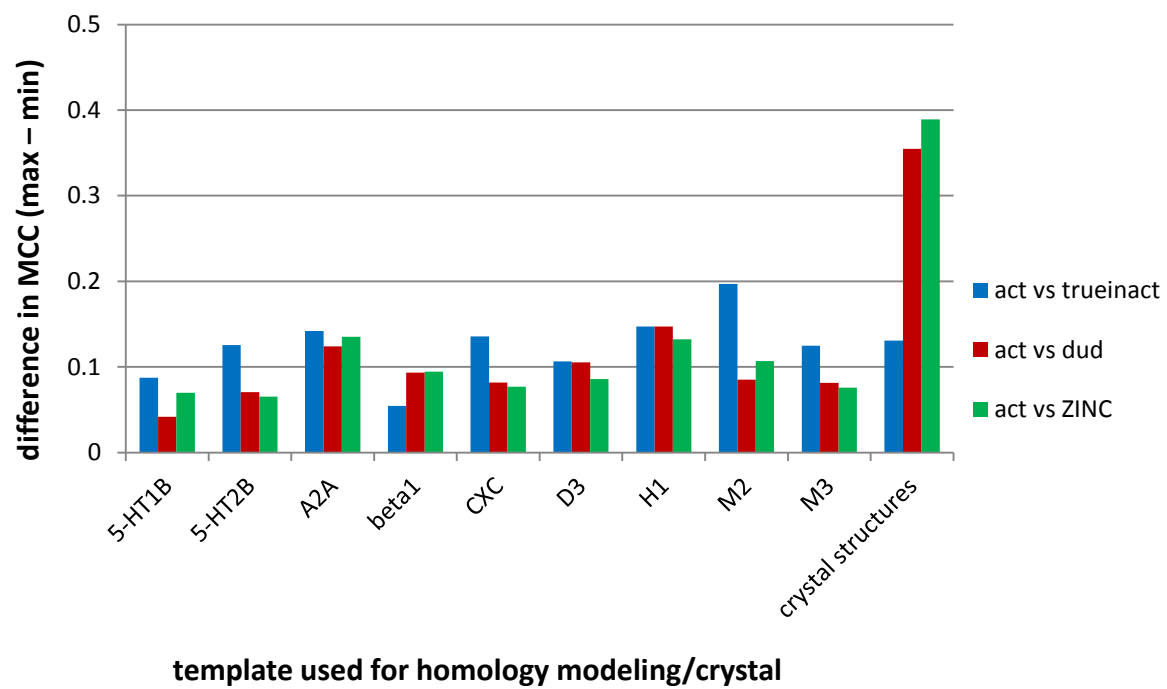

Supplement: Additional file 4: Figure S4. — Difference between the highest and the lowest MCC obtained for SIFt profiles construction for various numbers of conformations. The figure presents the scale of MCC changes associated with varying number of model conformations in the form of differences between the highest and the lowest. MCC values obtained for a given template/crystal structure. [file 13321_2015_62_MOESM4_ESM.pdf]
