# Supplementary material for: Multiple conformational states in retrospective virtual screening – homology models vs. crystal structures: beta-2 adrenergic receptor case study
Source: J Cheminform. 2015 Apr 9;7:13. doi: 10.1186/s13321-015-0062-x (PMC4420846; doi:10.1186/s13321-015-0062-x)

Figure S5. Comparison of the actives/true inactives classification efficiency for beta-2 homology models constructed on activated and deactivated M2R template.

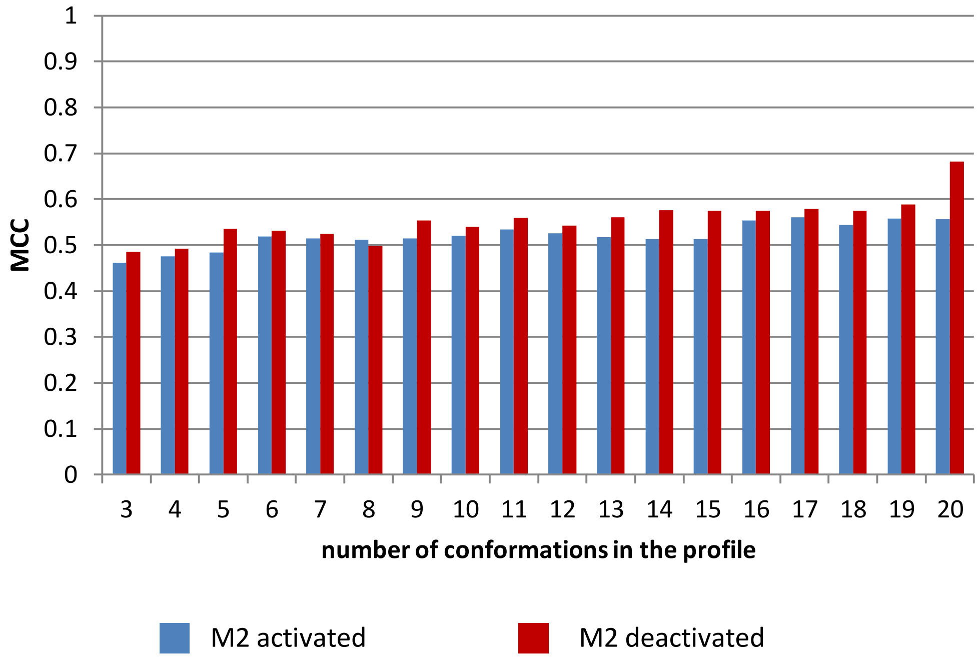

Supplement: Additional file 5: Figure S5. — Comparison of the actives/true inactives classification efficiency for beta-2 homology models constructed on activated and deactivated M2R template. The figure presents the differences between the results for beta-2 homology models that were constructed on crystal structure of the receptor with agonist (activated template) and on crystal structure in which the receptor was bound to antagonist (deactivated structure). [file 13321_2015_62_MOESM5_ESM.pdf]
